# Traumatic brain injury increases levels of miR‐21 in extracellular vesicles: implications for neuroinflammation

**DOI:** 10.1002/2211-5463.12092

**Published:** 2016-06-14

**Authors:** Emily B. Harrison, Colleen G. Hochfelder, Benjamin G. Lamberty, Brittney M. Meays, Brenda M. Morsey, Matthew L. Kelso, Howard S. Fox, Sowmya V. Yelamanchili

**Affiliations:** ^1^ Department of Pharmacology and Experimental NeuroscienceUniversity of Nebraska Medical CenterOmahaNEUSA; ^2^ Department of Cellular and Integrative PhysiologyUniversity of Nebraska Medical CenterOmahaNEUSA; ^3^ Present address: Albert Einstein College of Medicine1300 Morris Park AveBronxNY10461USA; ^4^ Present address: Medpace Reference Laboratories5365 Medpace WayCincinnatiOHUSA

**Keywords:** controlled cortical impact, exosomes, microglia, microRNA, neuroinflammation, secondary injury

## Abstract

Traumatic brain injury (TBI) is an important health concern and effective treatment strategies remain elusive. Understanding the complex multicellular response to TBI may provide new avenues for intervention. In the context of TBI, cell–cell communication is critical. One relatively unexplored form of cell–cell communication in TBI is extracellular vesicles (EVs). These membrane‐bound vesicles can carry many different types of cargo between cells. Recently, miRNA in EVs have been shown to mediate neuroinflammation and neuronal injury. To explore the role of EV‐associated miRNA in TBI, we isolated EVs from the brain of injured mice and controls, purified RNA from brain EVs, and performed miRNA sequencing. We found that the expression of miR‐212 decreased, while miR‐21, miR‐146, miR‐7a, and miR‐7b were significantly increased with injury, with miR‐21 showing the largest change between conditions. The expression of miR‐21 in the brain was primarily localized to neurons near the lesion site. Interestingly, adjacent to these miR‐21‐expressing neurons were activated microglia. The concurrent increase in miR‐21 in EVs with the elevation of miR‐21 in neurons, suggests that miR‐21 is secreted from neurons as potential EV cargo. Thus, this study reveals a new potential mechanism of cell–cell communication not previously described in TBI.

**Abbreviations**
CCI: controlled cortical impact
EV: extracellular vesicle
miRNA: microRNA
TBI: traumatic brain injury

Traumatic brain injury (TBI) is a leading cause of death and disability worldwide and current treatment strategies are limited 1, 2, 3. The damage caused by a TBI can be divided into the instantaneous primary mechanical injury and delayed secondary injury, which includes inflammation, neurochemical changes, and mitochondrial dysfunction 4. A robust inflammatory response is seen post‐TBI, including migration and activation of resident glia and recruitment of peripheral immune cells to the injury site 5. As in other types of injury, cell–cell communication is critical for regulating the immune response in TBI. Although there is a wide body of research examining the roles of cell–cell mediators such as cytokines and chemokines in TBI 6, the short duration of action, along with the complex and pleiotropic nature of these molecules make them difficult drug targets 4. In recent years, several important studies have found that extracellular vesicles (EVs) can influence cell–cell communication significantly 7, 8, 9. EVs are membrane‐derived vesicles that include vesicles that originate from the plasma membrane, exosomes derived from multivesicular bodies, and apoptotic bodies. These EVs serve as shuttles for cellular components between cells, carrying proteins, metabolites, lipids, mRNA, and miRNA 10. EV mRNA and miRNA can function within the recipient cell to alter protein expression 8, 11. Recent studies also show that miRNA carried within extracellular vesicles can trigger inflammatory responses and neuronal damage through pattern‐recognition receptors (PRRs) 9. While several experiments have shown effects of EV‐associated miRNAs (EV‐miRNA) on cells in culture, less is known about how the miRNA content of EVs is altered in disease conditions and whether or not EV‐miRNA has important pathophysiological roles. No studies so far have characterized EVs in the TBI brain. The goal of this study was to investigate changes in EV miRNA after a TBI. To accomplish this, we quantified levels of miRNA in EVs from mice 7 days after a TBI using next‐generation sequencing. Furthermore, *in situ* hybridization was performed to analyze the expression of miRNA in the brain.

## Methods

### Animals

Male C57BL/6 mice were obtained from Charles River Laboratories Inc. (Wilmington, MA, USA) and group housed in a 12 h light‐dark cycle and fed *ad libitum*. All procedures and protocols were approved by the Institutional Animal Care and Use Committee of the University of Nebraska Medical Center and conducted in accordance with the National Institutes of Health Guide for the Care and Use of Laboratory Animals.

### Controlled cortical impact

For all experiments, surgery was performed on mice 7–9 weeks old. Before surgery, mice were anesthetized using isoflurane and injected with 0.5 mg bupivacaine s.c. under the scalp, followed by an incision to access the skull. A 4‐mm craniotomy was performed midway between lambda and bregma on the left side. A Precision Systems and Instrumentation TBI‐0310 (Fairfax Station, VA, USA) was used to impact the exposed dura at a speed of 3.5 m·s^−1^ with a 200 ms dwell time and a depth of 1.0 mm. This CCI procedure was similar to that reported by others 12, 13. After the impact, Surgicel (Johnson & Johnson, Arlington, TX, USA) was used as a hemostatic agent, the skull placed over the brain and adhered with dental cement. The skin was secured with tissue clips. Sham surgery animals were anesthetized and placed in a stereotactic frame, injected s.c. with bupivacaine and an incision was made in the scalp. The skin was closed with tissue clips. Animals for each condition were chosen at random. Mice were sacrificed at indicated times after injury by isoflurane overdose followed by decapitation.

### Rotarod

Fifteen mice from each group were used for accelerating rotarod testing. Prior to injury all mice were trained for 3 days on the rotarod apparatus then mice were tested for 6 days, beginning 1 day after injury. On the first day, mice were acclimated to the rotarod for 5 min without movement. Mice were then tested three times a day with a minimum of a 30‐min interval between tests. The rotarod was set to accelerate from 0 to 35 rpm over a 2‐min interval and weight‐based sensors were used to detect latency to fall. For these experiments, a 7 cm diameter rotarod was used. This protocol was adapted from O'Connor *et al*. 14 The average latency to fall from the three trials of the last day of training are reported as day 0 and the average of three trials from each subsequent day was reported. Significance was determined using a two‐way ANOVA followed by Bonferroni *post hoc* tests.

### Tissue preparation for histology

Mice were anesthetized with isoflurane and sacrificed 7 days after injury by decapitation. Brains were removed and fixed in 4% paraformaldehyde overnight, paraffin embedded, and cut into 5‐μm coronal sections using a microtome. Staining was performed on sections between Bregma −1.3 and Bregma −2.5 mm, which included the lesion. Before staining, slides were warmed to 60 °C for 1 h and then allowed to cool. Slides were cleared with xylene then dehydrated through graded ethanol washes.

### Luxol fast blue staining

After hydration with 95% ethanol, slides were stained with filtered 0.1% luxol fast blue in a solution of 0.5% acetic acid at 60 °C overnight. On the next day, slides were rinsed in 95% ethanol followed by distilled water then differentiated in 0.05% lithium carbonate for one min and 70% ethanol for 1 min. Slides were counterstained with 0.5% cresyl violet for 30 min at 60 °C, rinsed in distilled H_2_O, and differentiated in 95% ethanol for 5 min. Coverslips were mounted using cytoseal (Thermo, Waltham, MA, USA). Immunostaining was performed on three biological replicates. Slide scanning was performed by the UNMC tissue sciences facility using a Ventana's Coreo Au Slide Scanner at 40× magnification.

### Immunohistochemistry

Staining was performed as described in Yelamanchili *et al*. 15. After hydration with a graded alcohol series, tissue was subjected to citrate antigen retrieval and washed with TBS. After blocking in a solution of 1% BSA and 3% NGS in PBS, tissue was incubated with primary antibody at a dilution of 1:500 for overnight at 4 °C. Antibodies used were anti‐GFAP (Dako Z0334, Carpinteria, CA, USA) and anti‐Iba‐1 (Wako 019‐19741, Richmond, VA, USA). On the following day, tissue was washed with TBS and incubated with 3% H_2_0_2_ in PBS for 10 min. After thorough washing, tissue was incubated with anti‐Rabbit secondary labeled with a peroxidase enzyme (ImmPRESS; Vector labs, Burlingame, CA, USA) for 1 h, rinsed, and developed with DAB Plus substrate system (Thermo) for 10 min. Tissue was then washed with TBS, stained with hematoxylin, and dehydrated. Coverslips were mounted with cytoseal (Thermo). Immunostaining was performed on three biological replicates.

### *In situ* hybridization

*In situ* hybridization was performed ,as described previously 16, with the addition of a 10 min peroxidase quenching step after the SSC washes using 3% H_2_O_2_ in PBS followed by TBS washes. In brief, after hydration and antigen retrieval, tissue was incubated with double DIG‐labeled LNA probes (Exiqon, Vedbaek, Denmark) overnight at 37 °C followed by SSC washes. Tissue was then incubated with antidigoxigenin‐POD, Fab fragments (Roche, Basel, Switzerland), 1:100, and primary antibodies at a concentration of 1:500 overnight at 4 °C. Antibodies used were anti‐GFAP (Dako Z0334) and anti‐Iba‐1 (Wako 019‐19741). Tissue was washed and incubated with secondary antibodies (Life Technologies, Carlsbad, CA, USA). TSA plus cyanine 5 (PerkinElmer, Waltham, MA, USA) was used for developing and 0.0001% DAPI was used as a nuclear counterstain. For mounting, coverslips proLong Gold Antifade Mountant (Life Technologies) was used. A Zeiss Observer.Z1 fluorescent microsope was used for imaging (Carl Zeiss, NY, USA).

### Extracellular vesicle isolation

Extracellular vesicles were isolated from brains, as described previously 17, using a method adapted from Perez‐Gonzalez *et al*. 18 Seven days after injury, animals were anesthetized with isoflurane and decapitated. Brains were extracted, cerebellum and brain stem were removed and the two hemispheres were separated. For each sample, four ipsilateral or four contralateral hemispheres were pooled. In total, 12 mice for each condition were utilized. This resulted in three pooled samples of each hemisphere from TBI mice and three pooled samples of each hemisphere from sham surgery controls, for a total of 12 samples altogether. After removal, tissue was snap frozen in liquid nitrogen and stored at −80 °C. Samples were thawed and digested in 20 units·mL^−1^ of papain in Hibernate A (Life Technologies), enzymatic digestion was stopped by the addition of cold Hibernate A, and the solution was further homogenized by trituration. Tissue fragments were removed by centrifugation and the supernatant passed through a series of successively finer filters (40, 5, and 0.2 μm). Remaining cell fragments were removed by centrifugation and the EV containing supernatant was submitted to several PBS washes followed by ultracentrifugation. A sucrose gradient was established using five concentrations of sucrose ranging from 0.25 to 2 m. The extracellular vesicle pellet was resuspended in the middle concentration (0.95 m), inserted into the gradient, and centrifuged at 200 000 ***g*** for 16 h at 4 °C. The extracellular vesicle containing central sections of the sucrose gradient were removed and resuspended to a total volume of 30 mL with PBS.

### RNA isolation and sequencing

The suspensions from the EV isolation were subjected to ultracentrifugation to pellet the EV. The pellets were then subjected to miRNA extraction using the mirVana miRNA Isolation Kit (Life Technologies) following the manufacturer's instructions. RNA samples were then sent to LC Sciences (Houston, TX, USA) for miRNA sequencing. Venny (http://bioinfogp.cnb.csic.es/tools/venny/) was used to create Venn diagrams.

### Electron microscopy

To validate the purity of EV isolation three hemispheres from three mice were pooled and snap frozen in liquid nitrogen and stored at −80 °C. EV isolation was performed as described above and the sample was submitted to the University of Nebraska Medical Center Electron Microscopy Core Facility to undergo microscopy by a FEI Tecnai G2 Spirit transmission electron microscope.

## Results

### Characterization of CCI

While the CCI model is commonly used in TBI research, the histopathology and behavioral deficits can vary dramatically with injury depth, species, strain, and age, as well as choice of controls. We chose a 1.0‐mm depth to model severe injury. While craniotomy only is a common control in the TBI field, it is associated with inflammation 19, 20. To avoid neuroinflammation caused by craniotomy, and to better control for peripheral injury and inflammation we used a peripheral injury control where an animals were given anesthesia, analgesia, and a scalp incision.

**Figure 1:**
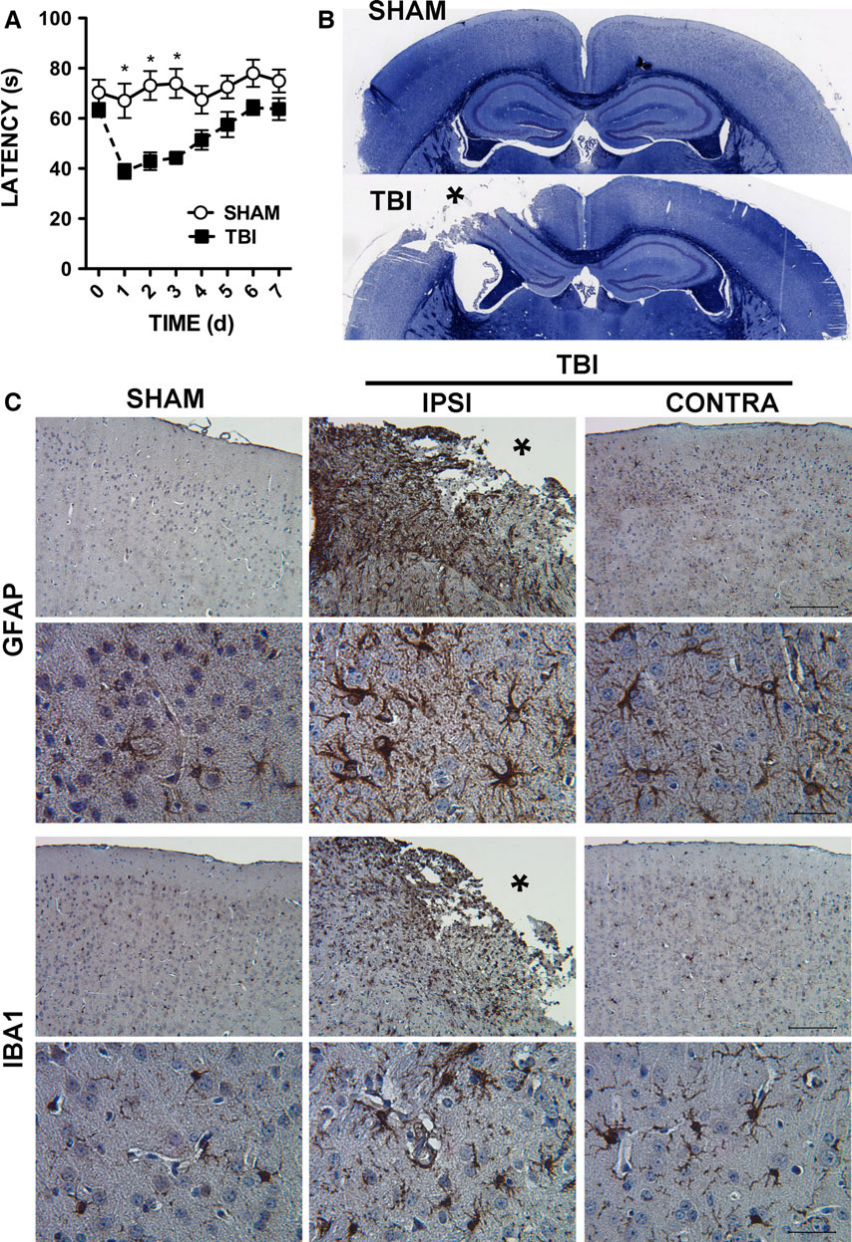
Characterization of CCI model. (A) Motor function in the week after CCI measured by rotarod. Sham controls and CCI mice were tested three times per day and trained for 3 days prior to injury. The average time to fall of three trials for each day is shown. Day 0 corresponds to the final day of training and Day 1 corresponds to the first day after CCI. The mean ± SEM from 15 animals are shown. A two‐way ANOVA was used to determine statistical significance. *P < 0.001. (B) Histology performed on mice 7 days after injury or sham surgery. Shown are representative images of three replicates. Luxol fast blue and cresyl violet staining of myelin (blue) and Nissl substance (purple) showing gross histology of the lesion, indicated by an asterisk, after CCI compared with the normal anatomy of the sham surgery control. (C) Iba‐1 and GFAP immunohisochemisty (IHC) staining for microglia and astrocytes, respectively, in the left cortex of sham controls (SHAM), and the ipsilateral (IPSI) and contralateral (CONTRA) cortices of animals after CCI (TBI). Images of the ipsilateral cortex show cortical tissue adjacent to the lesion site, lesion cavity indicated by an asterisk. Shown are representative images of three replicates. Original magnification 10× (bars = 100 μm), and 40× (bars = 25 μm).

Rotorod testing was then used to examine motor and vestibular function, as abnormalities are found after CCI 21. Before surgery, animals were trained on the accelerating rotarod apparatus for three trials a day for 3 days. On the last day of training, there was no difference in latency to fall between the groups randomly selected for CCI and sham surgery (Fig. 1A). Animals were tested daily, beginning 1 day after surgery. CCI impaired motor function 1, 2, and 3 days after injury, by day 4 post injury motor function recovered to the level of control. Peripheral injury controls did not show a decrease in motor function after sham surgery. Since rotorod deficits were resolved in 7 days after CCI, subsequent studies were performed at this time point.

Luxol fast blue staining was then performed to characterize the lesion site morphology and white matter damage 7 days after CCI or sham surgery (Fig. 1B). Sham animals showed no evident neuropathology; however, CCI‐induced cortical and hippocampal tissue loss on the injured side and enlargement of the lateral ventricle. Disruption of white matter tracts was clearly observable. The corpus callosum was disrupted and the fimbria was deformed.

Glial activation is a well‐recognized component of TBI pathophysiology 22. To examine the extent of glial activation 7 days after CCI, tissues were stained for GFAP and Iba1, markers of astrocytes and microglia, respectively (Fig. 1C). Levels of GFAP and Iba1 are both upregulated when glial activation occurs. As expected, staining for GFAP and Iba1 increased after TBI. In addition, microglial morphology shifted from ramified, resting microglia to a bushy, activated state in the injured hemisphere compared with sham controls. Activated microglia were also observed in the contralateral cortex after CCI, but to a lesser extent.

Overall, both motor impairment and neuropathology are consistent with descriptions of CCI by other groups. No changes in motor function or glial activation were seen in peripheral injury sham controls. Importantly, while motor deficits are resolved, glial activation is prominent 7 days after injury, we chose this time point to examine the role of EV miRNA in TBI‐induced neuroinflammation.

### Isolation and characterization of EV after CCI

**Figure 2:**
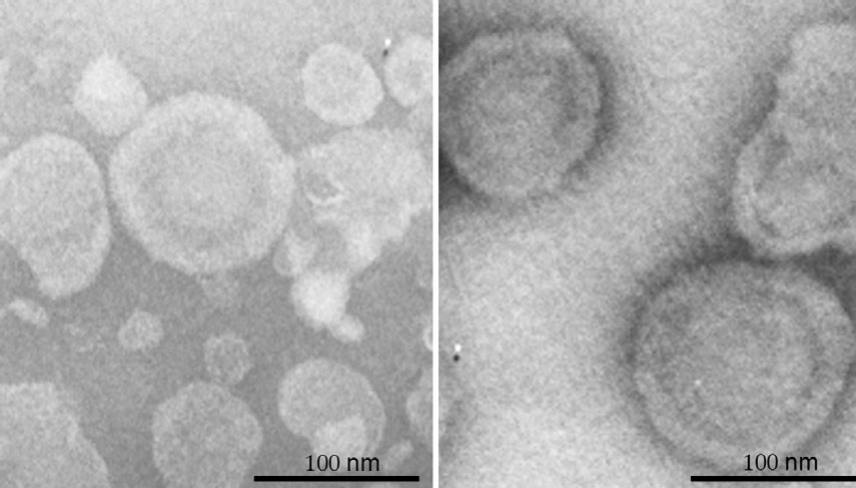
Characterization of EVs from brain tissue. Transmission electron microscopy of brain derived EVs showing a heterogeneous population of vesicles.

EVs were isolated from pooled brain tissue using differential centrifugation on a sucrose gradient. Transmission electron microscopy 23 was used to characterize vesicle size (Fig. 2). EVs isolated from mouse brain showed a heterogeneous‐sized population of EVs. Intact vesicles were present indicated by the characteristic “cup shape” created by the pooling of negative stain on top of the intact vesicle 10. These data indicate that intact, heterogeneous EVs were isolated from mouse brain.

### Sequencing of EV miRNA after CCI

**Figure 3:**
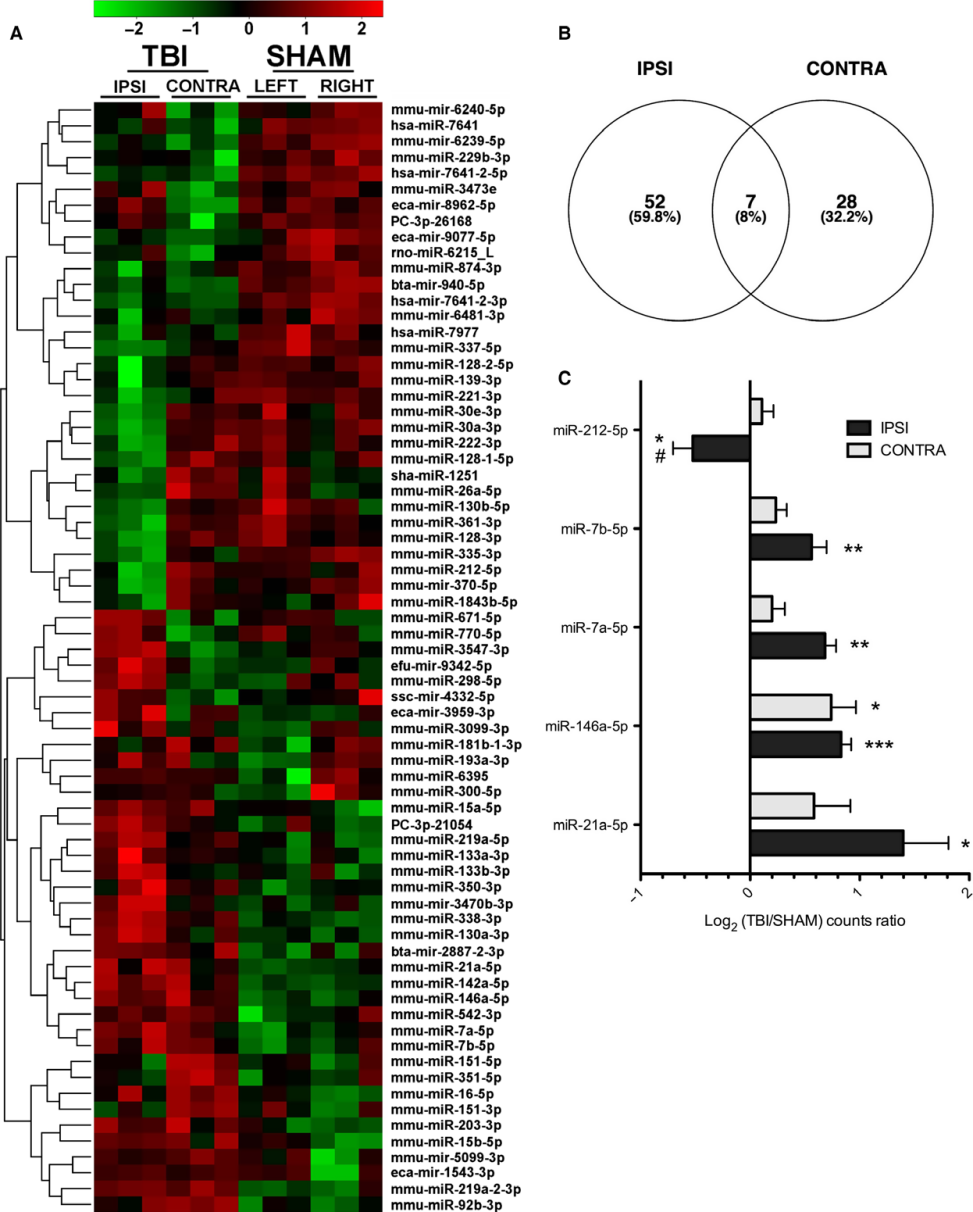
Sequencing of EV miRNA after CCI. (A) Heat map and hierarchical clustering depicting all differentially expressed miRNA (*P* < 0.05 by ANOVA). (B) Venn diagram showing differentially expressed miRNA in ipsilateral (IPSI) versus sham left and contralateral (CONTRA) versus sham right. Significance was determined by *T*‐test *P* < 0.05 (B). (C) miRNA that increase or decrease in the ipsilateral hemisphere (IPSI) relative to controls (log2 > 0.5) (C). Log2 values for the contralateral hemisphere (CONTRA) are also shown. The mean ± SEM from three replicates are shown. A one‐way ANOVA was used to determine statistical significance. *P < 0.05; **P < 0.01; ***P < 0.001.

Recent evidence has shown that EV miRNA can induce inflammation and neuronal damage 9, 17, 24, 25. Therefore, understanding changes in EV miRNA after CCI is relevant to TBI pathology. To quantify EV miRNA, EVs were isolated from the left and right hemispheres of animals 7 days after CCI or sham surgery. Then miRNA were purified from the EVs and sequenced. Brains from four conditions were used for this purpose: TBI ipsilateral (left) hemisphere, TBI contralateral (right) hemisphere, sham left hemisphere and sham right hemisphere. A heat map of all differentially expressed miRNA genes (*P* < 0.05 by ANOVA) is shown in Fig. 3A. Generally, miRNA clustered into those that increased or decreased in both the ipsilateral and contralateral hemispheres and those that increased only in the ipsilateral hemisphere. The largest number of differentially expressed miRNA (59) was found in the ipsilateral hemisphere from TBI relative to the corresponding sham hemisphere, followed by the TBI ipsilateral versus contralateral hemisphere (46). Only seven differentially expressed genes were common between the ipsilateral and contralateral hippocampi (Fig. 3B). Together this indicates that the ipsilateral hemisphere shows the most distinct set of differentially expressed EV miRNA, as would be expected considering the unilateral nature of the injury and glial activation seen by immunohistochemistry. To focus further examination, we calculated log2 values for differentially expressed miRNA and set a threshold of log2 = 0.5. On the basis of these criteria, we identified five differentially expressed genes, four upregulated and one downregulated in the ipsilateral hemisphere (Fig. 3C). Levels of miR‐212 were decreased in the ipsilateral hemisphere relative to corresponding sham and contralateral hemispheres. In contrast, miR‐7b, miR‐7a, and miR‐21 levels were all increased in the ipsilateral hemisphere compared with the corresponding sham hemisphere. Uniquely, miR‐146 was increased bilaterally in both the ipsilateral and contralateral hemispheres compared with sham. Of all the differentially expressed EV‐miRNA, miR‐21 showed the largest increase after CCI. Average counts for differentially expressed miRNA are shown in Table 1. The sequences in Table 2 show differentially expressed miRNA. Three of the five miRNA have GU‐rich sequences that are known to mediate TLR7/8 responses 26. In summary, CCI induced changes in miRNA associated with EVs, particularly in the ipsilateral hemisphere.

**Table 1:** Sequencing counts of miRNA significantly increased in the ipsilateral hemisphere (*P* < 0.05 with |log2 IPSA/Sham L)| > 0.5), average counts ± SD for three replicates, each pooled from three animals

miRNA	IPSI	CONTRA	SHAM L	SHAM R	IPSI/SHAM L	log2
miR‐21a‐5p	9283 ± 3860	6610 ± 2736	3275 ± 295	4179 ± 618	2.8	1.5
miR‐146a‐5p	3648 ± 413	3707 ± 1067	2042 ± 318	2163 ± 444	1.8	0.84
miR‐7a‐5p	11 393 ± 1411	9631 ± 1313	7057 ± 1115	8330 ± 910	1.6	0.69
miR‐7b‐5p	8582 ± 1411	8067 ± 932	5764 ± 642	6827 ± 1176	1.5	0.57
miR‐212‐5	2988 ± 666	4745 ± 594	4229 ± 115	4380 ± 931	0.71	−0.50

**Table 2:** Sequences of miRNA significantly increased in the ipsilateral hemisphere (*P* < 0.05 with |log2| > 0.5). GU‐rich sequences are shown in bold

miRNA	Sequence
miR‐21a‐5p	UAGCUUAUCAGACUGA**UGUUG**A
miR‐146a‐5p	UGAGAACUGAAUUCCAUGGGUU
miR‐7a‐5p	UGGAAGACUAGUGAUUU**UGUUG**U
miR‐7b‐5p	UGGAAGACUUGUGAUUU**UGUUG**U
miR‐212‐5p	ACCUUGGCUCUAGACUGCUUACU

### Localization of miR‐21 expression after CCI

**Figure 4:**
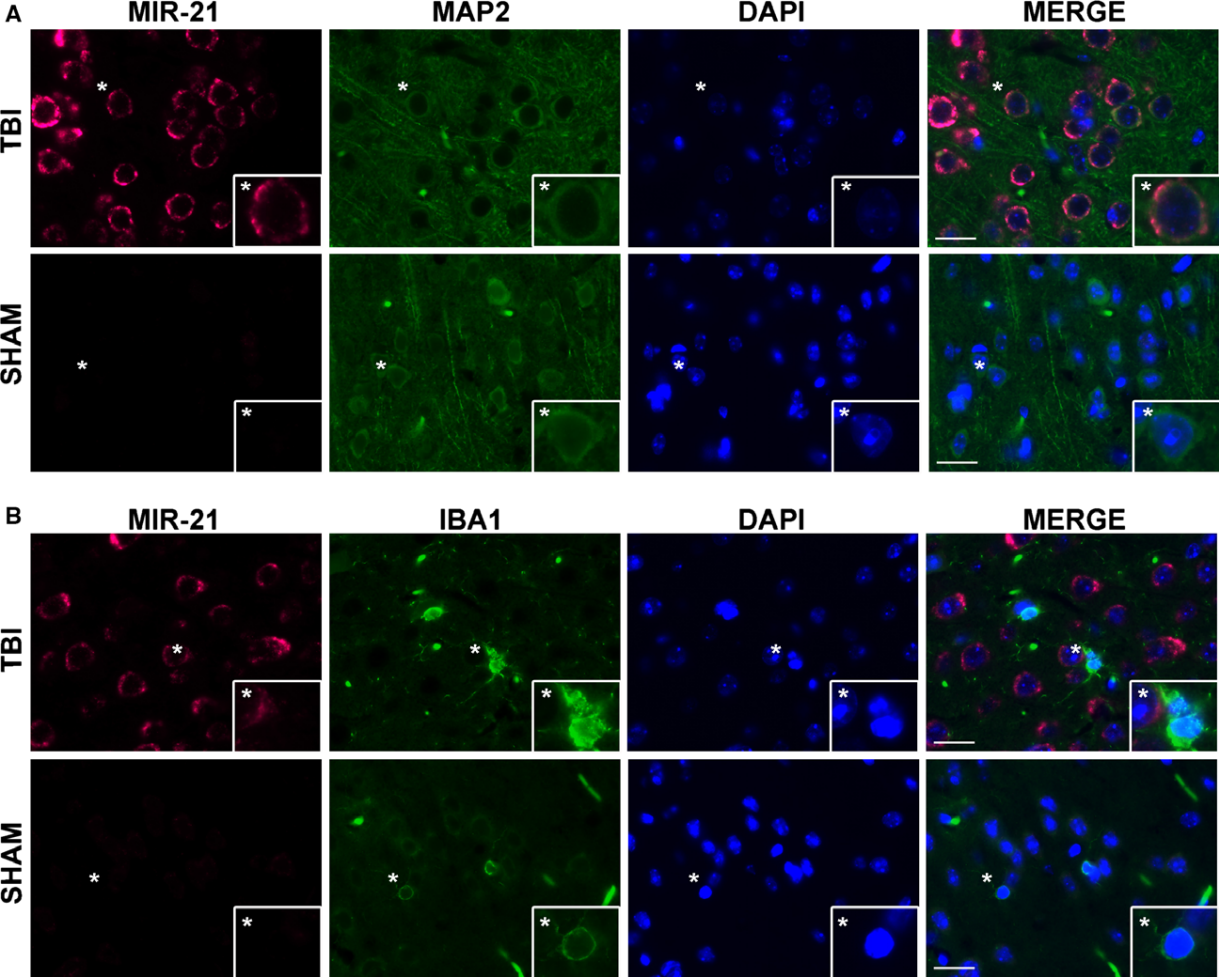
Localization of miR‐21 expression after CCI. Expression of miR‐21 was visualized by combined *in situ* hybridization and immunofluorescence. Staining for miR‐21 (magenta) and cell‐type markers (green), MAP2 (A) and Iba1 (B) were used to study neurons and microglia, respectively. Nuclei are stained with DAPI (blue). Original magnification 63× (bars = 100 μm), location of insets marked with an asterisk. Staining was performed in duplicate and representative images are shown.

Injury significantly increased EV miR‐21 in brain tissue. To investigate the cell‐type specific expression of miR‐21 after CCI, we performed combined immunofluorescence and *in situ* hybridization. Images were taken in the parietal cortex adjacent to the lesion, or lesion boundary. Expression of miR‐21 was higher in CCI animals than in the sham control (Fig. 4), which is in agreement with other reports 27, 28, 29. Costaining with MAP2, a cell‐type specific marker for neurons, showed colocalization with miR‐21, indicating that miR‐21 is highly expressed in neuronal cell bodies. In contrast, miR‐21 expression did not colocalize with microglial marker Iba‐1, suggesting that microglia are not the primary source of EV miR‐21. Intriguingly, activated microglia were found in immediate proximity to the miR‐21 positive neurons in the lesion boundary. While the colocalization does not prove that the origin of EV miR‐21 is strictly neuronal, since the majority of the staining is localized to neurons, we believe that neurons are primarily the source of EV miR‐21.

## Discussion

The major finding of this study is that TBI induces changes in EV‐associated miRNA in a rodent CCI model. Through miRNA sequencing, we found that miR‐21, miR‐146, miR‐7a, and miR‐7b all increased in the injured hemisphere relative to sham surgery control, while miR‐212 expression decreased. Of all the miRNAs tested, miR‐21 showed the largest fold change. Localization of miR‐21 expression through *in situ* hybridization was found overwhelmingly in MAP2 expressing neurons in the lesion boundary, suggesting that EV‐miR‐21 could be neuronal in origin and might mediate neuron‐glia signaling.

This is the first study to profile changes in brain EV miRNA after TBI. Several previous studies have identified changes in miRNA expression in brains from TBI models 27, 30, 31, 32, 33, 34. Of these studies, three reported a significant increase in miR‐21 relative to controls 27, 30, 34. Importantly, Meissner *et al*. 34 also showed that animals given a craniotomy alone without TBI did not show an increase in miR‐21 up to 12 h after injury. Furthermore, Sandhir *et al*. 29 showed increased miR‐21 expression as long as 7 days after TBI. The small quantity of EVs and EV‐associated RNA isolated from a single mouse brain hemisphere necessitates pooling of samples and limits sample size. However, the identification of changes in miR‐21 expression in EVs after TBI agrees with increases in whole brain tissue observed by others 27, 30, 34. Other than TBI, increased expression of miR‐21 has also been observed in many models of neuroinflammation and neuronal injury 35, 36, 37, 38, 39, 40, 41.

Generally, literature on miR‐21 in TBI supports a neuroprotective role for miR‐21. Treatment with a miR‐21 mimic improved disease outcomes in rats after CCI 28. Also, overexpression of miR‐21 reduces neurotoxicity in an *in vitro*, stretch model of TBI through miR‐21 targeting of PTEN 42. Increasingly, miR‐21 is recognized as an important molecule in neuronal injury 43. The neuroprotective and regenerative effects of miR‐21 have been observed in models of stroke 44, axotomy 38, and neurodegeneration 45. Additionally, miR‐21 has roles in glial responses to injury. In spinal chord injury, miR‐21 reduces hypertrophy of astrocytes, reducing glial scar formation 39, whereas in experimental stroke, miR‐21 targets FasL in microglia reducing microglia‐mediated neuronal death 46. Despite the potential benefits of miR‐21 expression in neuronal injury, there are also drawbacks. For example, in HIV‐associated neurocognitive disorders, elevated expression of miR‐21 contributes to neuronal dysfunction by increasing the potassium channel activity and targeting MEF2C, an important neuronal transcription factor 40. Elevated miR‐21 also contributes to neuropathic pain in nerve injury 37. Nevertheless, this yin‐yang role of miR‐21 makes it an interesting target of study in neuronal injury and inflammation. Here, for the first time, we have identified that miR‐21 can also be associated with EVs in TBI.

This study particularly profiled EV‐miRNA from the brain tissues of CCI injured mice. Previously, Patz *et al*. 47 also characterized EV‐miRNA after TBI, but in the cerebrospinal fluid (CSF) of patients. Our miRNA sequencing did not recapitulate the miRNA profile found in this previous study. This difference could be due to several factors, such as the use of CSF versus brain tissue, and patients versus a controlled experimental model. Outside of the TBI field, profiling of EV‐miRNA from neurons and brain tissue has been performed. Interestingly, exosomes secreted by prion‐infected neurons show higher levels of miR‐21 than exosomes from uninfected neurons *in vitro* 48. This supports the theory that neurons increase the release of miR‐21 in EV as a response to stress. Our group and others have studied the role of miRNA in exosomes or extracellular vesicles in HIV‐associated neurocognitive disorders (HAND), conditions strongly linked with neuroinflammation 49. One study indicated that comorbid HIV infection and opiate abuse can increase miR‐29 packaging in brain EV, which in turn downregulates the important neuroprotective molecule PDGF 50. Another study showed that in HAND, EV‐miR‐21 is increased and mediates neurotoxicity by binding to TLR7 and causing necroptosis 17. As more groups profile miRNA signatures of EVs, a clearer picture will form of which miRNA are common to neuronal injury or inflammation and which are specific to disease state.

The release of miRNA in EVs is thought to have two possible effects on target cells. The first mechanism occurs when the EV either fuses with the cell membrane or endosomal membrane to release its contents into the cytosol 11. Mature miRNA in the cytosol can bind to target mRNA and decrease their translation 7. The second mechanism of EV miRNA action is through binding of pattern‐recognition receptors in the endosomal compartment, primarily toll‐like receptor 7/8 (TLR7/8) 9. TLR7/8 recognizes ssRNA, and elicits an antiviral response as part of the innate response to viral pathogens 51. Several groups have reported that miRNA with EV can stimulate TLR7/8, but the outcome of miRNA binding to TLR7/8 is highly dependent on cell type. In immune cells, such as macrophage and microglia, TLR7/8 binding elicits a proinflammatory response, including secretion of TNFα 9. Alternatively, TLR7/8 binding in neurons is toxic and can lead to cell death or synaptic loss 17, 24, 25. The binding of miRNA to TLR7/8 is dependent on GU‐rich sequences 26, as miR‐21 has such a GU‐rich sequence it is a strong stimulator of TLR7/8 9, 17, 24. Recent studies have identified EV‐miR‐21 specifically as both proinflammatory 9 and neurotoxic 25. Our studies on SIV encephalitis showed that not only was miR‐21 elevated in EV from encephalitic brains, but also that EV‐miR‐21 induced necroptosis in neurons through TLR7 17. Therefore, the increase in EV miR‐21 reported in this study has important pathophysiological implications for TBI. Whether miR‐21 in EV leads to translational regulation or TLR7/8 stimulation in recipient cells after TBI is not addressed in this study and will be an important question for future research.

Aside from miR‐21, we identified three other miRNA with increased levels in EVs, miR‐146, miR‐7a, and miR‐7b. Increased expression of total brain miR‐146a was reported Lei *et al*. 30 in a CCI model of TBI. Interestingly, our previous studies in SIV encephalitis also found increases in EV‐miR‐146 17. It is known that inflammatory stimuli such as lipopolysaccharide (LPS) induce miR‐146 52. Interestingly, knockout studies in mice have proven miR‐146 to be an important anti‐inflammatory miRNA 53. Even more importantly, miR‐146 within exosomes can act functionally to reduce inflammation in recipient cells 54. Therefore, it is possible that in TBI miR‐146 within exosomes could reduce neuroinflammation. Relatively a little is known about miR‐7a and b compared with miR‐146 and miR‐21. However, some *in vitro* data suggest that miR‐7 can be neuroprotective 55. The expression of miR‐7 is relatively brain‐specific 56. Interestingly, miR‐7a and b both contain a GU‐rich element identical to that found in miR‐21 (UGUUG) indicating that they may also be ligands for TLR7/8 (Table 2). We found one miRNA, miR‐212, down‐regulated in EV after TBI. Downregulation of miR‐212 has been found in several brain diseases 57. These include anencephaly 58, schizophrenia 59, and Alzheimer's disease 60. Together these studies hint that deregulation of miR‐212 may be pathological in the brain.

## Conclusion

We report here the first miRNA profile of brain exosomes in TBI. Differential expression of five miRNA was found between EVs from CCI‐injured brain versus uninjured controls. Of the differentially expressed miRNA, miR‐21 showed the highest increase in EVs of the injured brain. Interestingly, increased levels of miR‐21 were found in neurons of the injury boundary zone near reactive microglia. Further studies need to be done to test whether neuronal derived EV miR‐21 transmigrates into adjacent microglia and activates them.

This work shows that TBI induces changes in EV miRNA, which likely has important consequences for cell–cell signaling and in the disease progression in TBI.

## Author contributions

SY, MK, EH, and HF conceived and designed the project. EH, CH, BL, BM Morsey, and BM Meays collected and analyzed the data. EH, CH, and SY interpreted the data. EH and SY wrote the manuscript. SY, MK, HF, CH, BL, BM Morsey, and BM Meays all contributed to the editing of the manuscript.
